# Supplementary material for: Analgesic Effect of a Novel Intravenous Ibuprofen-Low-Dose Tramadol Combination: A Multimodal Approach to Moderate-to-Severe Postoperative Dental Pain
Source: Pharmaceutics. 2025 Sep 24;17(10):1248. doi: 10.3390/pharmaceutics17101248 (PMC12566731; doi:10.3390/pharmaceutics17101248)
Supplement: Supplementary file 1 [file pharmaceutics-17-01248-s001.zip › supp files/Supp file Ap1_Synopsis_ protocol.pdf]

DOUBLE-BLIND, RANDOMISED, PLACEBO-CONTROLLED, PILOT STUDY TO EVALUATE THE COMPARATIVE EFFICACY OF IBUPROFEN COMBINED WITH DIFFERENT DOSES OF TRAMADOL AND TRAMADOL 100 MG ADMINISTERED INTRAVENOUSLY IN PATIENTS WITH MODERATE TO SEVERE PAIN AFTER DENTAL SURGERY

PROTOCOL CODE: FMLD-FEBETRADI-PILOT-43_FIII

EUDRA-CT: 2018-001412-30

SYNOPIS

Sponsor:<br>Laboratorios FARMALIDER S.A.<br>c/La Granja N1<br>28108 Alcobendas, Madrid - Spain	Name of active ingredient:<br>Ibuprofen (arginate)/Tramadol HCl
Title:<br>Double-blind, randomised, placebo-controlled, pilot study to evaluate the comparative efficacy of ibuprofen combined with different doses of tramadol and tramadol 100 mg administered intravenously in patients with moderate to severe pain after dental surgery	
Protocol Code:<br>FMLD-FEBETRADI-PILOT-43_FIII	
EudraCT:<br>2018-001412-30	
Coordinating Investigator:<br>Dr. Antonio Portolés Pérez<br>Clinical Pharmacology Department, Hospital Clínico San Carlos	
Analysis site<br>Laboratorios Unilabs<br>C/ Juan Esplandiu 15<br>28007 Madrid	
Ethics Committee:<br>Clinical Research Ethics Committee of Hospital Clínico San Carlos	
Objectives:<br>*Primary objective:*<br>The preliminary assessment of analgesic efficacy measured by the Visual Analogue Scale (VAS) at 6 hours after administration of a single dose of the fixed-dose combination of ibuprofen (arginate) 400 mg with two dose levels of intravenous tramadol versus tramadol 100 mg IV, placebo-controlled.<br><br>*Secondary objectives:*<br>• To estimate the measure and variability of the analgesic effect of each combination of ibuprofen (arginate) and tramadol IV vs placebo.<br>• To estimate the measure and variability of the analgesic effect of tramadol 100 mg IV vs placebo.<br>• To assess the safety and tolerability of each of the medications or combinations used, to improve the safety profile of the patients.	
Study design:<br>Double-blind, randomized, multicentre, phase IIb-IIIa, pilot clinical trial with active comparator and placebo-controlled to assess the efficacy and safety of ibuprofen combined with different doses of tramadol in patients with moderate to severe pain after dental surgery.	
Inclusion criteria:<br>1. Patients who give their written informed consent and are willing to fulfil all visits and scheduled procedures required by the protocol.<br>2. Patients ≥ 18 years old.<br>3. Body weight >50 and < 110 kg.<br>4. Medical history and physical examination without clinically relevant anomalies depending on the study and at the discretion of the investigator.<br>5. Scheduled for outpatient surgical removal, under local anaesthesia, of at least two third molars, at least one of them lower, and at least one of them impacted requiring bone removal.<br>6. Patients who accept not to take analgesics except those defined by the protocol as rescue medication during the treatment period, up to 7 hours after administration of the study medication.<br>7. With moderate-severe pain (VAS ≥55 mm) for the first 4 hours after the end of surgery.	

Exclusion criteria:

1. Patients with a history of allergy or hypersensitivity to the study medication, rescue medication or any other nonsteroidal anti-inflammatory (NSAIDs), opiates or acetylsalicylic acid, or any of its excipients.
2. A history of asthma, bronchospasm, acute rhinitis, nasal polyps, hives or angioneurotic oedema.
3. A history of peptic ulceration, gastrointestinal disorders, gastrointestinal bleeding or other active bleeding.
4. A history of moderate to severe renal, hepatic or cardiac failure.
5. Haemorrhagic diathesis or other bleeding disorders.
6. Epilepsy.
7. Crohn's disease or ulcerative colitis.
8. A history of drug or alcohol dependence.
9. History of any disease or disorder that, at the investigator's discretion, could pose a risk to the patient or alter the results of the study (eg. patients with acute pain of any other origin or location at the time of surgery).
10. Patients who have had complications during surgery lasting more than 1 hour and requiring re- anaesthesia (after reaching the appropriate level of anaesthesia).
11. Patients who have taken any analgesic (including prescription and over the counter) during 48 hours prior to surgery, or within 5 days before in the case of COX-2 inhibitors.
12. Patients unable to abstain from alcohol, psychotropics or sedatives (e.g. benzodiazepines) or other medicines that should not be administered due to the risk of interactions for 48 hours before initiation of surgery and within 12 hours of administration of the study medication.
13. Patients who have received an experimental drug or used an experimental medical device within 30 days prior to screening process.
14. Pregnant or breastfeeding women.
15. Patients who cannot to meet the study requirements or who in the opinion of the investigator should not participate.

Treatment groups:
Group 1: Ibuprofen (arginate)/tramadol HCl 400/37.5 mg
Group 2: Ibuprofen (arginate)/tramadol HCl 400/75 mg
Group 3: Tramadol 100 mg
Group 4: Placebo. 100 ml
A single dose will be administered over 30 minutes.

Duration of treatment:
Single dose infused in 30 minutes.

Study endopints:
Primary efficacy variable:
Pain intensity at 6 hours measured through the VAS, adjusted for baseline VAS

Secondary efficacy variables:
Pain intensity difference (PID_{7h}), sum of pain intensity differences ($SPID_{7h}$), pain relief (PAR_{7h}), total pain relief ($TOTPAR_{7h}$), patient responder rate, rate of rescue medication use and time until administration of rescue medication for each treatment group.
Safety variables: Vital signs (body temperature, heart rate and blood pressure), laboratory tests and adverse event assessment.

Sample size:
Based on previous confidential data from studies conducted by the sponsor, a sample size of 72 subjects would be sufficient to evaluate the analgesic efficacy of CDF IBU/TRA compared to tramadol HCl 100 mg and placebo, assuming 80% power and a two-sided significance level of 0.05. Taking into account an estimated 20% rate of serious protocol violations and screening failures, the sample size could be increased to 112.

Statistical Analysis:
The collection of patients' personal data will be conducted in hard copy format, with subsequent storage

of these records in a file that is compliant with the prevailing data protection regulations. Information pertaining to the study will be stored in an electronic database.
In accordance with current legislation, an external monitor will oversee the clinical trial to ensure compliance with GCP and other relevant regulations. Once the final patient has completed their visit, the database will be finalised. After it has been closed, the data will be downloaded for statistical analysis.
The general characteristics of the sample will be described. This will include demographic characteristics and other variables included in the electronic Case Report Form. Qualitative variables will be expressed as a percentage and 95% confidence interval (where possible), and quantitative variables will be expressed as the mean and standard deviation. If the distribution is not normal, they will be expressed as the median and interquartile range. An exploratory analysis will be performed to search for outliers and missing values. If missing values are detected in the dependent variables, the use of multiple imputation techniques will be considered.
The primary variable of the study is pain intensity assessed using the VAS scale. To this variable, an ANCOVA will be performed to compare the VAS at 6 hours between the different treatment groups, adjusted for baseline VAS. This main analysis will be performed on the ITT population (intention-to-treat), therefore, information on all recruited patients who received the treatment will be included, except for those who withdrew their consent to participate in the trial and those recruited patients who did not meet the inclusion criteria and did not receive any intervention.
Descriptive techniques and an analysis of variance (ANOVA) model will be used for the analysis of PID, SPID, PR, and TOTPAR, where appropriate. Post hoc analysis will be performed where possible. To analyse the time to rescue medication administration, a survival analysis (time to event) will be performed using Cox regression to evaluate the differences between groups. Information on safety and tolerance will be presented through a descriptive analysis.
