# Supplementary material for: Analgesic Effect of a Novel Intravenous Ibuprofen-Low-Dose Tramadol Combination: A Multimodal Approach to Moderate-to-Severe Postoperative Dental Pain
Source: Pharmaceutics. 2025 Sep 24;17(10):1248. doi: 10.3390/pharmaceutics17101248 (PMC12566731; doi:10.3390/pharmaceutics17101248)
Supplement: Supplementary file 1 [file pharmaceutics-17-01248-s001.zip › supp files/supp file Appendix 2_study team.pdf]

Appendix 2

Febetradi study Team

**Study site**	**Name**
Hospital Clínico San Carlos - IDISSC	Alejandro Jiménez-Ortega
Hospital Clínico San Carlos - IDISSC	Angela Rivas
Hospital Clínico San Carlos - IDISSC	Daniel Lozano
Hospital Clínico San Carlos - IDISSC	Diana Uribe
Hospital Clínico San Carlos - IDISSC	Farzin Falahat
Hospital Clínico San Carlos - IDISSC	M Carmen Martín-Cordón
Hospital Clínico San Carlos - IDISSC	Manuel de-Pedro-Marina
Hospital Clínico San Carlos - IDISSC	Natalia Pérez Macías
Hospital Clínico San Carlos - IDISSC	Percy Chávez
Hospital Clínico San Carlos - IDISSC	Sonia Herrero
Hospital Universitario de La Princesa	Ana L Capote-Moreno
Hospital Universitario de La Princesa	Dolores Ochoa
Hospital Universitario de La Princesa	Gina Mejia-Abril
Hospital Universitario de La Princesa	Manuel Román
Hospital Universitario de La Princesa	Tamara Garcia
Hospital Universitario de La Princesa	Verónica Escorial-Hernández
Hospital La Paz. IDIPAZ	Eduardo Vazquez
Hospital La Paz. IDIPAZ	Irene García-García
Hospital La Paz. IDIPAZ	Javier González
Hospital La Paz. IDIPAZ	Jorge Noguera
Hospital La Paz. IDIPAZ	Jose Luis del Castillo
Hospital La Paz. IDIPAZ	Laura Vitón
Hospital La Paz. IDIPAZ	Lucía Díaz-García
Hospital La Paz. IDIPAZ	María José Morán
Hospital La Paz. IDIPAZ	Vega Mauleón
Hospital Ramón y Cajal	Itziar de-Pablo-López-de-Abechuco
Hospital Ramón y Cajal	Mónica Aguilar-Jiménez
Fundación jiménez Díaz	Francisco Perez-Flecha
Fundación jiménez Díaz	Guillermo García-Serrano
Fundación jiménez Díaz	Leonardo Trujillo
Fundación jiménez Díaz	Lourdes Maniegas
